# Current Approaches Including Novel Nano/Microtechniques to Reduce Silicone Implant-Induced Contracture with Adverse Immune Responses

**DOI:** 10.3390/ijms19041171

**Published:** 2018-04-12

**Authors:** Shin Hyuk Kang, Chanutchamon Sutthiwanjampa, Chan Yeong Heo, Woo Seob Kim, Soo-Hong Lee, Hansoo Park

**Affiliations:** 1 Department of Plastic and Reconstructive Surgery, Chung-Ang University Graduate School of Medicine, Seoul 06974, Korea; kangshinhyeok@hotmail.com (S.H.K.); kimws@cau.ac.kr (W.S.K.); 2 School of Integrative Engineering, Chung-Ang University, Seoul 06974, Korea; pupura_chan@hotmail.com; 3 Department of Plastic and Reconstructive Surgery, Seoul National University Bundang Hospital, Seongnam 13620, Korea; lionheo@gmail.com; 4 Department of Biomedical Science, CHA University, Kyunggi 13488, Korea

**Keywords:** silicone breast implant, capsular contracture, foreign body reaction, T-cell immunity, ALCL, nano/microtechniques

## Abstract

Capsular contracture, which is the pathologic development of fibrous capsules around implants, is a major complication of reconstructive and aesthetic breast surgeries. Capsular contracture can cause implant failure with breast hardening, deformity, and severe pain. The exact mechanisms underlying this complication remain unclear. In addition, anaplastic large cell lymphoma is now widely recognized as a very rare disease associated with breast implants. Foreign body reactions are an inevitable common denominator of capsular contracture. A number of studies have focused on the associated immune responses and their regulation. The present article provides an overview of the currently available techniques, including novel nano/microtechniques, to reduce silicone implant-induced contracture and associated foreign body responses.

## 1. Introduction

Silicone has been used widely in medicine for the last 70 years, with the first implant placed in humans in 1946 for duct repair during biliary surgery [1]. Since then, silicone-based materials have been used extensively in implants in humans, including pacemakers, cardiac valves, hydrocephalic shunts, aesthetic implants, orthopedic implants, nerve conduits, and dental implants. The aesthetic application of silicone is mainly in plastic and reconstructive surgery; it has also been used to correct congenital deformities or defects after reconstructive surgeries following trauma or cancer. 

The most frequent use of this material is in silicone breast implants. Since 1961, when Dow Corning along with the Houston-based cosmetic surgeons Tom Cronin and Frank Gerow developed the first silicone breast implants, which were rubber sacs filled with viscous silicone gels, silicone mammary implants have been associated with a number of risks and complications, which has significantly limited their application. The formation of a constrictive fibrotic capsule around the implant with a concurrent foreign body reaction post-implantation, known as capsular contracture, is experienced by up to 50 percent of patients after breast augmentation and reconstruction [2,3,4,5]. Clinically, significant breast capsular contracture is characterized by excessive constrictive fibrotic capsule formation that leads to firmness, distortion, and displacement of the breast implant [6]. Baker et al. developed a clinical classification system of capsular contracture after breast implant surgery, specifically: grade І capsular contracture of the augmented breast feels as soft as an unoperated breast. Grade ІІ capsular contracture is minimal; the breast is less soft than an unoperated breast; the implant can be palpated but is not visible. Grade ІІІ capsular contracture is moderate; the breast is firmer; the implant can be palpated easily and may be distorted or visible. Grade IV capsular contracture is severe; the breast is hard, tender, and painful, with significant distortion. The capsule thickness is not directly proportional to the palpable firmness, although some relationship may exist [7].

Capsular contracture remains the major reason underlying patient dissatisfaction and additional subsequent surgeries [8,9]. Additional surgeries are required in severe cases, but these procedures are complex, challenging, and unpredictable. Therefore, secondary procedures cannot guarantee a successful outcome without recurrence. The surface type and biomaterial are very important in regulating the inflammatory foreign body reaction in the surrounding tissue. The foreign body reaction is a natural tissue reaction that occurs after inserting an implant. There is no clear conclusive theory to date, but the consensus is that the immune system plays a very important role in the development of capsular contracture. The current understanding of capsular contracture is that it involves a complex combination of bacterial contamination in pockets and other related factors that stimulate inflammation around the implant, leading to a proliferation of fibroblasts along with collagen deposition and contracture [10,11]. However, the detailed mechanisms underlying capsular contracture remain unclear.

## 2. Anaplastic Large Cell Lymphoma (ALCL)

Anaplastic large cell lymphoma (ALCL) is a very rare breast implant-associated T-cell lymphoma that is CD30^+^ and anaplastic lymphoma kinase (ALK) negative [12,13,14]. This disease is now widely recognized and there is an increased public awareness of the association between breast implants and the development of ALCL, a rare form of non-Hodgkin’s lymphoma after warnings were released from the U.S. Food and Drug Administration on 26 January 2011 [15]. ALCL CD30^+^ occurs due to the activation and abnormal proliferation of T lymphocytes [15,16]. ALCL is classified into cutaneous and systemic forms and the expression of the *ALK* gene characterizes the ALCL into subtypes and determines the prognosis. The morphology and cytology of breast implant-associated ALCL (BIA-ALCL) are similar to those of ALK-negative systemic ALCL. However, ALK-negative systemic ALCL has a poor prognosis, while breast implant-associated ALK-negative ALCL typically has a benign course with better prognosis [17]. These cases present as late-onset seroma with the swelling or mass of the unilateral breast [15,18]. These clinical symptoms are not consistent with the clinical symptoms of both cutaneous and systemic ALCL. Therefore, there is agreement that breast implant-related ALCL should be classified as a separate clinical entity [14,19,20,21].

**Figure 1:**
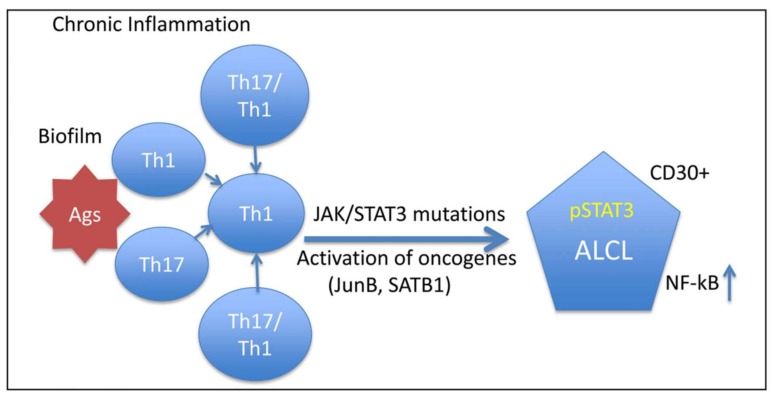
Hypothesis: Breast implant-associated anaplastic large cell lymphoma (ALCL) is caused by persistent T-cell immune reactions to chronic stimulation from bacterial antigens (Ags) and subsequent genetic events. Arrow means progression of immune responding T lymphocytes to BIA-ALCL [25].

To date, there has been no population-based estimation of the incidence of ALCL in women with breast implants in the United States. De Jong et al. reported an epidemiological study of ALCL in women with breast implants in the Netherlands [12]. They calculated an incidence of ALCL of 0.1 to 0.3 per 100,000 women per year based on an estimate of 100,000 to 300,000 Dutch women with breast implants [12]. Antonella et al. reported that the estimated incidence of the Italian BIA-ALCL cases reported to 2015 as 2.8 per 100,000 patients [22]. Recent studies have found the incidence of breast implant-associated ALCLs has increased with the clinical use of implants with larger textured surface areas [23], which induce an excessive inflammatory response and chronic antigenic stimulation due to bacterial infection (Figure 1) [24,25]. Most confirmed cases of breast implant-associated ALCLs have occurred in women with textured breast implants. However, cases of BIA-ALCLs in patients with smooth type breast implants have also been reported [26,27,28]. According to the global adverse event reports of breast implant-associated ALCL published by Srinivasa et al. in 2017, the textured type was significantly more prevalent than the smooth type (50% versus 4.2%; *p* = 0.0001), but its use was unknown or not reported in 44.6% cases [29].

Silicone implants induce a specific local immune reaction involving activated T-helper (Th1/Th17) cells that results in fibrosis, which is facilitated by the increased production of profibrotic cytokines due to the decrease in local T regulatory cell functions [24,30]. Miler and Anderson [31] reported that interleukin (IL)-1 is generated by macrophages following exposure to silicone. IL-1 acts as an immunochemical message to T-lymphocytes that a foreign substance has appeared and initiates immune activation. There have been many reports of specific T-lymphocyte proliferation in silicone implant capsules of augmented women [30,32,33]. Intracapsular T cells predominantly produce IL-17, IL-6, IL-8, transforming growth factor-beta1 (TGF-β1), and interferon-gamma, suggesting a Th1/Th17-weighted local immune response [30]. Promising novel therapies for preventing inappropriate stimulation of T cell responses and complications are being studied. In this review, we provide an overview of the current techniques aimed at reducing silicone implant-induced contracture with concurrent foreign body responses, including novel nano/microtechniques. 

## 3. Approaches to Reducing Adverse Immune Responses

### 3.1. Drugs

#### 3.1.1. Systemic Drugs

Breast infections following implant-based breast reconstructive surgery can form persistent biofilms and develop capsular contracture [34]. Furthermore, delaying the postoperative prophylactic use of antibiotics following implant-based breast reconstruction increases the risk of surgical-site infection, reoperation, and reconstructive failure [35]. There is no global consensus on the duration of antibiotic prophylaxis after breast reconstruction. Standardized definitions of antibiotic regimens are needed. The use of leukotriene antagonists (LTAs) to treat capsular contracture was reported in 2002 [36,37] and many studies have since demonstrated the benefits of these LTAs, including softening of breasts and reduction in severity of capsular contracture, using either montelukast (Singulair) [38] or zafirlukast (Accolate) [39,40]. The protective role of LTAs is based on their antagonist effect on TGF-β1 [41]. The pharmacological action of LTAs involves competitive binding with cysteinyl-leukotrienes receptor type 1 (Cys-LT1) [41]. The cysteinyl-leukotrienes facilitate TGF-β1 production, which results in fibroblast proliferation and fibrosis, suggesting that these Cys-LT1 receptor antagonists reverse fibroblast remodeling and fibrosis [42]. Treatment with Cys-LT1 receptor antagonists can decrease IL-6, IL-10, IL-13, and TGF-β1 levels, all of which are elevated in fibrotic lungs [43]. However, some studies indicate the effects of leukotriene receptor antagonists are anecdotal [39,44]. In 2007, the angiotensin-converting-enzyme inhibitor Enalapril was reported to decrease the expression of fibrotic mediators, TGF-β1, inflammatory markers, monoclonal antibodies to ED1 (CD68) and collagen III, and the periprosthetic fibrosis process [45].

In 2008, a short-term study on the use of Pirfenidone (PFD) to prevent capsular contracture after the installation of silicone implants in rats reported that PFD clearly reduced the capsular thickness of the surrounding submammary tissue, fibroblast-like cell proliferation, and recruitment of inflammatory cells [46]. PFD is a wide spectrum anti-fibrotic agent that modulates various inflammatory cytokines and has promising effects in the prevention and regression of many fibrotic diseases [47,48,49]. In the 2008 study, the total collagen content in the PFD-treated group was 50% lower and fibroblasts had a 45% less activated phenotype than in the control group. Furthermore, expression of the *TGF-β* and *collagen 1* genes was also reduced by 85% and 60%, respectively, in rats after oral administration of PFD compared to the control group [46]. A controlled clinical trial published in 2013 reported the effectiveness of oral PFD in breast capsular contracture [50]. This open, controlled, prospective, pilot clinical trial was conducted to assess the efficacy of 1800 mg oral PFD per day for 6 months. However, side effects, such as photosensitivity, rash, and itching sensation, nausea, diarrhea, and abdominal discomfort, have been reported [46].

Oral colchicine, a drug commonly used in the treatment of gout, appears to decrease the severity of capsular contracture in an animal model [51]. Colchicine is a well-known drug that inhibits inflammation. It inhibits cell migration and proliferation through disruption of microtubule polymerization by binding to a cytoskeletal protein [52]. The most common side effect of colchicine is gastrointestinal distress, such as diarrhea and vomiting, which limits its use and can lead to stoppage of therapy [53].

Baker et al. found that alpha-tocopherol (vitamin E) reduces the incidence of spherical constriction that occurs around breast implants [54]. Vitamin E is the main lipid-soluble antioxidant that inhibits the accumulation of peroxides, protects cells from damage by free radicals, and contributes to the stability and integrity of biological membranes. Vitamin E shows beneficial effects on various inflammatory diseases [55,56,57]. Based on this, the early administration of vitamin E reportedly aided in reducing fibrous capsule contracture following breast augmentation [54]. In addition, the prophylactic use of vitamin E is effective for capsule contracture in patients who receive adjuvant radiotherapy after reconstructive surgery with implants [58].

Recently, Kang et al. reported that oral administration of a synthetic tryptophan metabolite, Tranilast, reduced capsule formation in a rabbit model [59]. Tranilast inhibits the release of chemical mediators from inflammatory cells such as monocytes/macrophages [60], neutrophils [61], and lymphocytes [62,63] that are involved in capsule formation. It also inhibits the release of related cytokines that regulate T-cell immunity; it also inhibits TGF-β1 release, as well as collagen synthesis [59].

#### 3.1.2. Topical Application 

The incidence of capsular contracture is decreased by the use of povidone-iodine [64,65] and antibiotic irrigants [66] during breast implant surgery. In addition, because a significant increase in both infections and seromas, which lead to capsular contracture, was observed in patients not treated with topical antibiotics, some studies also support the use of topical antibiotics during cosmetic breast surgeries [67]. The use of povidone-iodine irrigation reduces Baker class III/IV capsular contracture and is not associated with implant rupture or increased deflation of saline breast implants [64,65]. However, due to the low methodologic quality of related studies, recommendations for perioperative povidone-iodine irrigation as standard procedure are limited [64,65].

Topical use of botulinum toxin type A prevents capsule formation around silicone implants, possibly by blocking TGF-β1 signaling and interrupting differentiation of fibroblasts into myofibroblasts. In 2016, botulinum toxin type A was reported to reduce capsule formation around silicone implants and inhibit differentiation of fibroblasts into myofibroblasts through the TGF-β/Smad signaling pathway in vivo [68,69]. The occurrence of hematoma is one of the various causes of capsular contracture. Spyropoulou et al. showed that hyaluronidase, which causes reabsorption of bleeding, can reduce capsular contracture in a rabbit model [70]. In addition, clinical use of hyaluronidase in subglandular or submuscular pockets before implant placement was reported to reduce capsule formation [71]. However, the authors did not perform a histological and statistical analysis. Primarily used as a cytotoxic chemotherapeutic, 5-fluorouracil only decreases cellular metabolism and blocks protein synthesis at lower concentrations. Therefore, it may be used to prevent capsule formation around silicone breast implants if it is loaded onto slow-releasing carriers, such as gelatin block [72]. Along with 5-fluorouracil, mitomycin C, which is used as an antitumor agent and antibiotic, can inhibit DNA synthesis through functional alkylation of double helix crosslinking, as well as inhibiting RNA and protein synthesis. Therefore, the topical application of mitomycin C may prevent fibroblast proliferation and collagen synthesis [73].

Xiaflex, a collagenase from the bacterium *Clostridium histolyticum*, can degrade human capsular contracture tissue ex vivo. However, skin perforation and adequate drug distribution within the implant pocket are issues with Xiaflex that need to be addressed prior to clinical use [74,75]. Short-term in vitro studies have demonstrated a dose-dependent decrease in capsule thickness from skin injections of Xiaflex. Reductions in collagen subtypes 1, 2, and 3 concentrations, as well as an up-regulation of profibrotic and inflammatory markers, were observed following Xiaflex treatment [76]. In 2016, Fischer et al. [74] described a dose-dependent reduction in human capsule contracture tissue ex vivo in a Xiaflex-treated group. Only collagen type 4 remained after degradation, which can act as a neo-capsule/acellular tissue matrix. In 2017, Fischer et al. [75] reported significant reductions in capsule thickness and collagen density following Xiaflex treatment in a study of the long-term effects of using this collagenase to treat capsular fibrosis in vivo. Low expression of collagen subtypes, as well as significant down-regulation of TGF-β3, was also detected in the group injected with Xiaflex. Anti-adhesion agents can reduce capsule thickness and the myofibroblast ratio [77]. Moreover, leukotriene receptor antagonists reduce silicone-induced peri-implant capsule formation in both white rabbit and rat models [78,79].

In 2015, Park et al. reported the acute suppression of TGF-β with the local and sustained release of synthetic tryptophan metabolite (Tranilast) in the formation of fibrous capsules around silicone implants in a rat model [80]. Finally, the slow distribution of prednisolone by liposome localization reportedly decreases fibrous capsule thickness around textured silicone implants [81] and the introduction of triamcinolone acetonide into implant pockets influences early capsule formation and reduces capsular contracture in a rabbit model [81,82].

In 2011, Sconfienza et al. reported that ultrasound-guided topical application of triamcinolone acetonide 40 mg to peri-implant capsules in patients undergoing breast implant surgery reduced capsule thickness and patient discomfort [83]. Zeplin et al. reported the potential of topical halofuginone application in decreasing the foreign body response [84]. Halofuginone is an alkaloid derived from *Dichroa febrifuga*, a flowering plant in the family Hydrangeaceae. It inhibits Smad3 phosphorylation in the TGF-β signaling pathway, which results in a marked inhibition of *collagen α I* gene expression [85]. Halofuginone has been used to inhibit various fibrotic disorders in vivo [86,87,88]. Submuscular implantation of implants applied with halofuginone in animal models can decrease CD68^+^, histocytes, TGF-β, fibroblasts, collagen type I and type III, and capsular thickness [84].

Systemic uses and the topical use of colchicine and vitamin E have also been reported [89,90]. In an in vivo study, insertion of silicone implants applied with colchicine resulted in less inflammatory infiltrate, no myofibroblasts, and random and disorganized collagen fibers around the implants compared to the control group, which might result in prevention of capsule formation [89]. Topical application of vitamin E and croton oil to reduce pseudocapsules around the prosthesis has also been studied [90]. Individuals with topically applied vitamin E and croton oil showed thicker pseudocapsules and more marked cellular infiltrate than individuals who received an intramuscular injection of vitamin E [90].

Ng et al. [91] had studied the effect of local delivery of nicotine from the implant surface on the reduction of capsular contracture formation. There was no significant difference in capsular thickness between the nicotine-treated group and the control group. However, significant differences in angiogenesis were observed, which may potentially be useful for the fabrication of other biomaterials [91]. Li et al. [92] demonstrated the ability of medical chitosan to reduce capsule formation around breast implants by blocking the signaling pathway of tissue inhibitor of metalloproteinases (TIMPs) in vivo. At 4, 8, and 12 weeks following surgery, both capsular thickness and the expression of TIMP-1 and TIMP-2 were significantly lower in the medical chitosan-treated group than in the control group [92].

### 3.2. Materials

#### 3.2.1. Combined with Autologous Tissues (Fat Grafts)

Autologous fat grafting has drawn increasing attention and gained widespread acceptance for improving the outcomes of breast reconstruction. Fat grafting to the breast has been widely used since 2008 when the 1987 moratorium imposed by the American Society of Plastic Surgeons was reversed [93]. However, fat grafting has natural limitations in the breast due to its soft nature. Therefore, “composite breast surgery”, a combination of the classic implant technique and simultaneous management of overlying soft tissue with fat grafting, was introduced as a new paradigm. Breast augmentation that simultaneously uses implants and fat is a more powerful and versatile approach and achieves a synergistic outcome [94]. Moreover, multiple clinical studies have demonstrated that autologous fat grafts reduce the incidence of postoperative complications, including capsular contracture [95,96]. Additionally, an animal study suggested autologous fat transfer could be used to treat capsular contracture by neovascularization of the tissue around the implant [97]. However, this technique remains controversial for use in aesthetic breast surgery. 

#### 3.2.2. Combined with Acellular Dermal Matrix

The acellular dermal matrix (ADM) has recently emerged as a potential tool for surgical prevention of capsular contracture. ADMs are immunologically inert materials that minimize capsule formation in animal models and clinically. Animal studies have shown that implants completely wrapped in the allograft display decreased inflammation, myofibroblast cell proliferation, and capsule thickness [98]. Moreover, ADM envelopes can decrease radiation-induced inflammatory changes and pseudoepithelium formation in an irradiated rat model. These findings suggest a slower progression to capsular contracture [99]. In a 13-year long-term study, Salzberg et al. [100] reported the low cumulative incidence of capsular contracture with ADM-assisted direct-to-implant reconstruction, even in irradiated breasts. In this study, capsular contracture was considered an early event occurring within the first 2 years after reconstruction because longer follow-up durations found no increase in incidence. These findings suggest that ADM may truly prevent the development of capsular contracture. Since then, surgeons have modified the ADMs with strategically placed fenestrations to increase support to the implant within a rapidly expanding pocket and ultimately improve the aesthetic result. Despite these manipulations, fenestrated ADMs decrease capsular contracture rates in a manner similar to non-fenestrated matrixes [101]. In addition to its prevention of capsular contracture, ADMs have also been useful for the treatment of capsular contracture. Cheng et al. [102] reported a novel technique using complete coverage of implants with ADM to treat capsular contracture. 

### 3.3. Surface Type

#### 3.3.1. Smooth and Textured Surfaces

Implant surface texture, which acts as the interface between the device and the body tissue, has a dramatic influence. Therefore, much attention has been given to implant surface textures, i.e., smooth versus textured, and their impact on the development of capsular contracture [103]. Ersek [104] reported that texture can alter the host interface and induce a multiplanar conformation of collagen fibers, which results in thinner and more pliable capsules that are more resilient and less likely to contract. Liu et al. [105] studied the formation of capsular contracture after implantation of smooth or textured silicone breast prostheses in a meta-analysis. Smooth breast implants were significantly more associated with capsular contracture than were textured implants. Other long-term studies and meta-analyses have reported significantly more capsular contracture in primary subglandular breast augmentations using smooth implants than ones using textured implants [3,106,107]. In a pig model, Minami et al. [108] found that the thickness of the capsular contracture in the group with the smooth implants was thicker than the group with the textured implants (mean capsule thickness around smooth vs. textured implants measured 270 days post-implantation was 2040 μm vs. 1281 µm, respectively). Abramo et al. applied the parallelogram law to linear vectors drawn within concavities in the textured surface to achieve their resultant contraction vector [109]. In this study, the pore diameter and depth of the texture displayed significant effects on fibrous capsule formation. Textured implants with large open-pore diameters (250–350 µm, and 600–800 µm) and depth (40–100 µm) resulted in normal breast firmness. In contrast, textured implants with a small open-pore diameter (70–150 µm) and depth (10–760 µm, 150–200 µm) resulted in very long resultant vectors over the fibrous capsule and increased breast firmness. The implants with a macrotextured surface (open-pore diameter 600–800 µm; depth 150–200 µm) led to the formation of vectors of different lengths and directions, which significantly reduced the risk of fibrous capsular contraction. Capsular contracture can be reduced, although not eliminated, with textured implants. However, other complications have appeared with the use of textured breast implants. Double capsules and late seromas have not been observed for smooth saline or smooth silicone gel breast implants, but have been observed for aggressively textured implants [110]. Mechanical shear stress applied to an immature periprosthetic capsule has been linked to the formation of capsules [111]. Moreover, implants with larger textured surface areas have a significantly higher risk of breast implant-associated ALCL [23]. Therefore, to overcome complications related to the texture of implant surfaces, nano/microtechnologies were recently introduced. These innovations have brought enormous changes to breast surgery.

#### 3.3.2. Surface Modification Using Nano/Microtechnology

**Figure 2:**
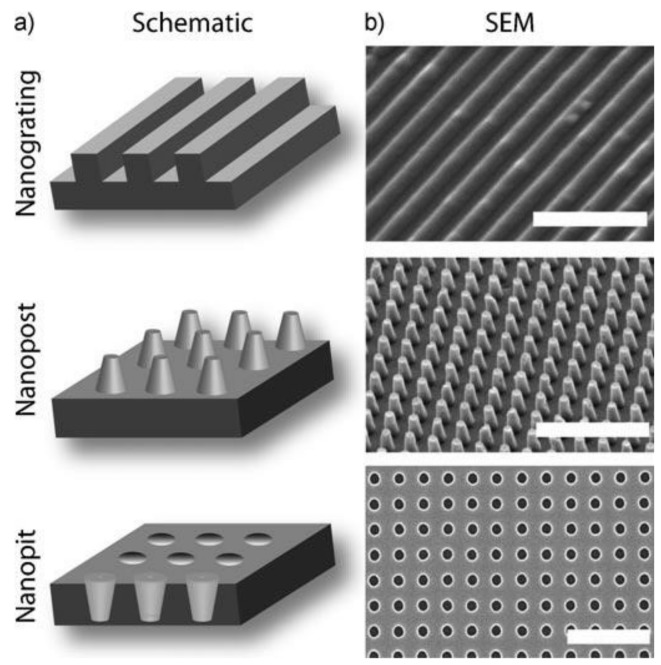
Schematic illustrations (**a**) and SEM images (**b**) of representative nanotopography geometries. Nanograting (scale bar = 5 µm), nanopost (scale bar = 5 µm), and nanopit array (scale bar = 1 µm) [114].

The geometries of nano/microtopographies in the extracellular matrix (ECM) contribute to cell interactions and signaling [112]. Surface nano/microtopographies influence cell polarization, alignment, migration, attachment, adhesion, proliferation, and morphology naturally at the nano/micro level as the cells react naturally to surrounding structures [112,113,114]. T-cell migration, which is considered to play an important role in capsular contracture and ALCL, is also highly dependent on interactions between cells and the implant in the ECM [115]. The latest nano/micro-engineering techniques have enabled the analysis and fabrication of native tissue topography. There are three basic nanotopography geometries: nanogratings, nanoposts, and nanopits (Figure 2) [114].

**Figure 3:**
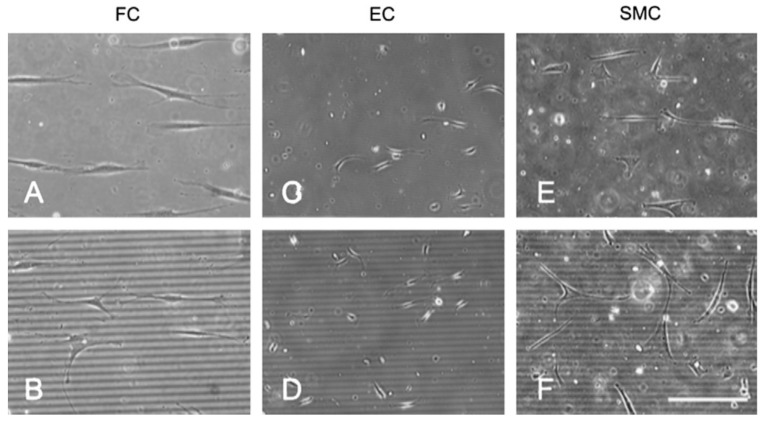
Human fibroblasts (FCs), endothelial cells (ECs), and smooth muscle cells (SMCs). (**A**,**C**,**E**) The grooves on substrates with 2-μm-wide grooves with a depth of 200 nm; (**B**,**D**,**F**) The grooves on substrates with 10-μm-wide grooves.Deep (200 nm) grooves with 2 and 10 μm wide grooved substrates are evident in phase-contrast images taken 24 h after seeding on polydimethylsiloxane (PDMS) substrates (scale bar = 150 μm) [116].

**Figure 4:**
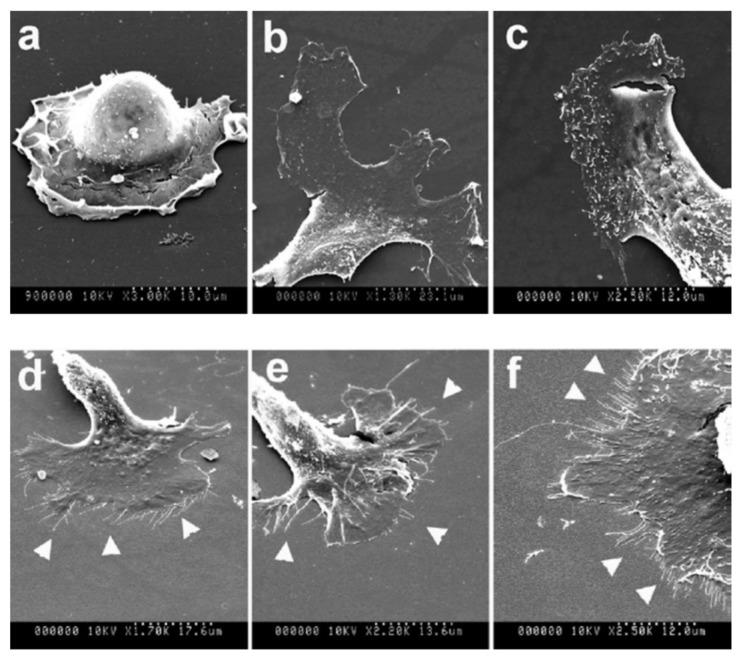
Flat (**a**–**c**) and 10-nm-high island (**d**–**f**) substrates. Fibroblasts on the latter substrate display fringed lamella with numerous filopodia (arrowheads) [117].

**Figure 5:**
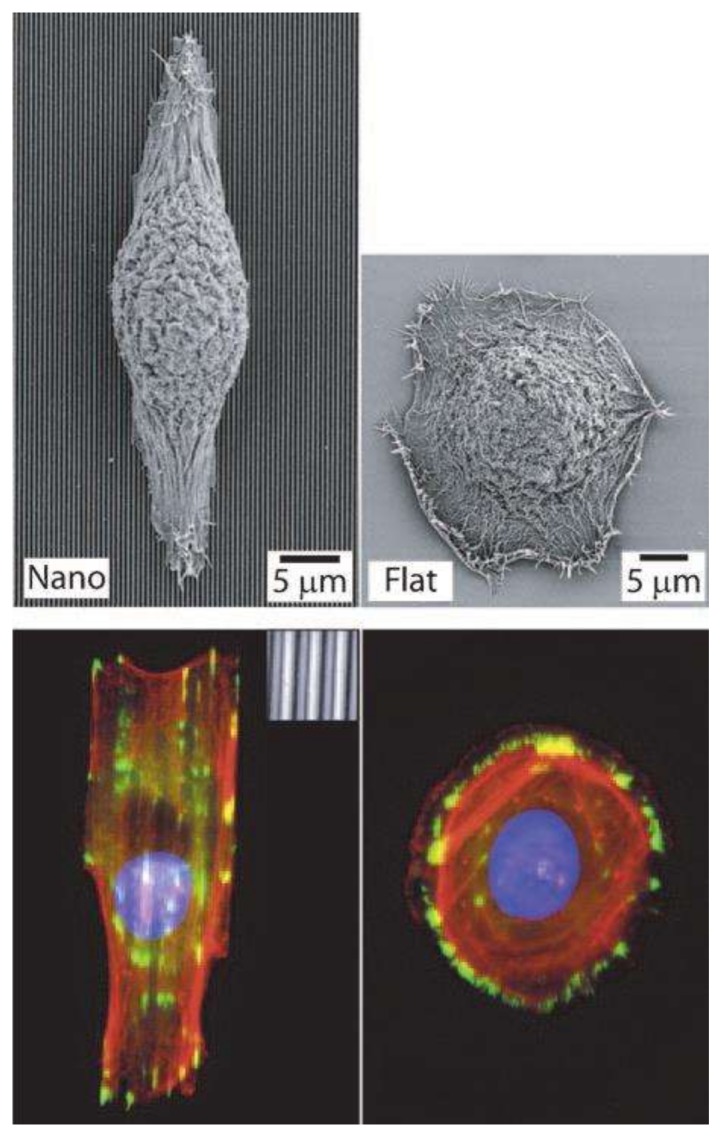
Epithelial cell response to nanograting. Fluorescence (**bottom**) and SEM (**top**) images. Arrangement and alignment along the grid axis. Reproduced with permission from the Company of Biologists [114].

**Figure 6:**
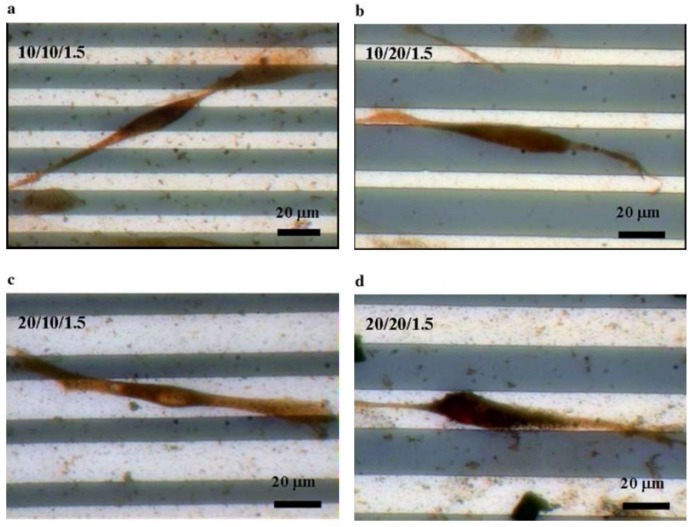
Schwann cells on a patterned silicon substrate (anti-S-100 stain, observed using optical microscopy). (**a**) 10/10/1.5 μm; (**b**) 10/20/1.5 μm; (**c**) 20/10/1.5 μm; and (**d**) 20/20/1.5 μm [118].

The reactions of various cells to substrates fabricated using this technique have been studied with human fibroblasts (FCs), endothelial cells (ECs), smooth muscle cells (SMCs), and Schwann cells (Figure 3, Figure 4, Figure 5 and Figure 6) [114,116,117,118].

Biela et al. [116] studied the effect of surface structures on the shape and alignment of FCs, ECs, and SMCs after seeding them on polydimethylsiloxane (PDMS) substrates for 24 h. Most cells aligned along the grooves on substrates with 2-μm-wide grooves with a depth of 200 nm (Figure 3A,C,E); cell alignment was much lesser on substrates with 10-μm-wide grooves (Figure 3B,D,F) [116]. 

Dalby et al. [117] observed filopodia of FCs cultured on a substrate with 10-nm-high islands. Figure 4 shows low-magnification SEM images of cells on control (planar) and 10-nm-high island substrates. FCs with clear, well-spread lamellae were observed on both the control and island substrates. However, a greater number of filopodia were observed on the substrate with 10-nm-high islands than on the control substrate [117].

Bettinger et al. [114] reported that the impact on cell geometry was the most noticeable effect of nanotopography on cell function. The response of various types of cells, such as FCs, ECs, SMC, stem cells, epithelial cells, and Schwann cells, upon seeding on nanogratings was to commonly align and elongate in the direction of the grating axis [114]. Figure 5 demonstrates the alignment and elongation of epithelial cells [114]; Figure 6 shows the same response by Schwann cells [118]. Hsu et al. [118] studied the alignment of Schwann cells on microgrooves and found that their width/spacing was also important for determining cellular alignment (Figure 6). When the grooves were narrower than 1.5 μm, Schwan cells were attached to the spaces between the grooves rather than aligning within the grooves themselves. This response was observed most prominently on 10/10 μm, 10/20 μm, and 20/10 substrates and least on the 20/20 μm substrate (Figure 6) [118].

**Figure 7:**
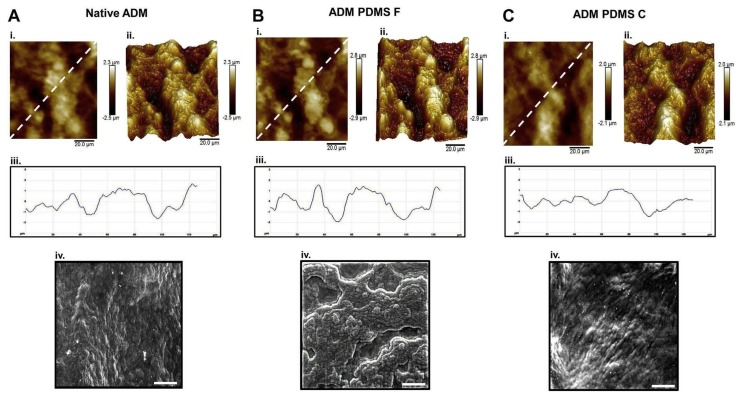
(**A**) Native acellular dermal matrix (ADM); (**B**) ADM PDMS fabricated surface (ADM PDMS F); and (**C**) ADM PDMS cast surface (ADM PDMS C) (i) Two-dimensional atomic force microscopy and (ii) three-dimensional images. (iii) Section profiles of two-dimensional and (iv) SEM image (scale bars (iv) = ×1000 magnification) [119].

**Figure 8:**
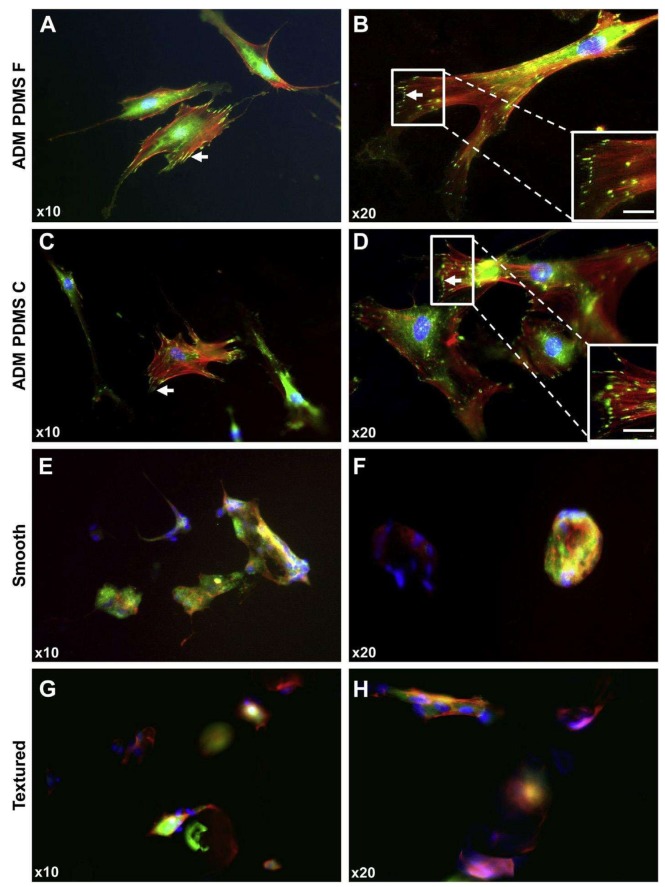
Immunofluorescence images of breast-derived fibroblasts (BDFs) on various PDMS surfaces. (**B**,**D**) BDFs on ADM PDMS formed focal contacts (white arrows) with substrates (white scale bars = ×40 magnification); (**A**,**D**) Fibroblasts on ADM PDMS F and C surfaces attached and spread. The representative “spindle-like” morphology is shown; (**E**,**F**) Conversely, BDFs on smooth silicone implant surfaces had a poor cell-substrate attachment; (**G**,**H**) BDFs on textured implant surfaces also had poor cell attachment with some focal contact formation. Moreover, cells appeared got trapped in the deep troughs between the steep nodules [119].

The development, fabrication, and functional evaluation of new biocompatible polydimethylsiloxane (PDMS) surfaces with various nano/microtopographical features enable favorable foreign body reactions. Kyle et al. [119] reported that the nano/microscale features of ADM can be successfully replicated with PDMS using a new three-dimensional grayscale fabrication technique (Figure 7 and Figure 8). 

Figure 7A–C shows that both the PDMS fabricated surface (ADM PDMS F) and the ADM PDMS cast surface (ADM PDMS C) techniques were able to reproduce ADM topography and roughness of PDMS accurately. However, the ADM PDMS C technique reproduced the surface features most reliably (Figure 7C(i–iv)) [119]. Figure 7A(iv),B(iv) show SEM images, which demonstrate that both techniques were able to elucidate features down to 10 s of nanometers in scale [119].

Figure 8A–D shows immunofluorescence images with focal staining of vinculin in breast-derived fibroblasts (BDFs) on ADM PDMS surfaces [119]. Kyle et al. [119] have reported that the formation of focal contacts at the tips of F-actin filaments is characteristic of focal adhesions, indicating that BDFs on ADM PDMS surfaces are able to form a stable attachment with the underlying biomimetic topography, and subsequently spread to develop typical fibroblast “spindle-like” morphology. Figure 8E, F shows immunofluorescence staining of BDFs on smooth implant surfaces [119]. The cells showed a round morphology containing diffuse and non-specific vinculin staining with no focal contact formation. BDFs aggregated on the smooth implant surfaces and were often seen to bind to each other rather than forming focal contacts with the underlying implant surface topography [119]. On the other hand, BDFs on textured implant surfaces showed mostly diffuse and non-specific staining of vinculin and minimal focal contact formation, with a significant number of cells appearing to be trapped in deep troughs between the nodules (Figure 8G,H) [119]. 

**Figure 9:**
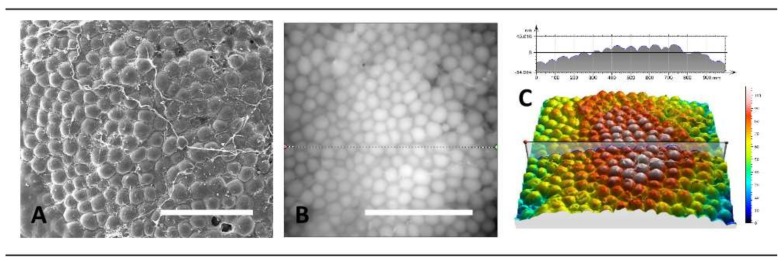
(**A**) Native breast adipose tissue surface (SEM image, scale bars = 500 µm); (**B**) grayscale laser confocal height images; and (**C**) three-dimensional projection of image B cross-sectional profile [120].

**Figure 10:**
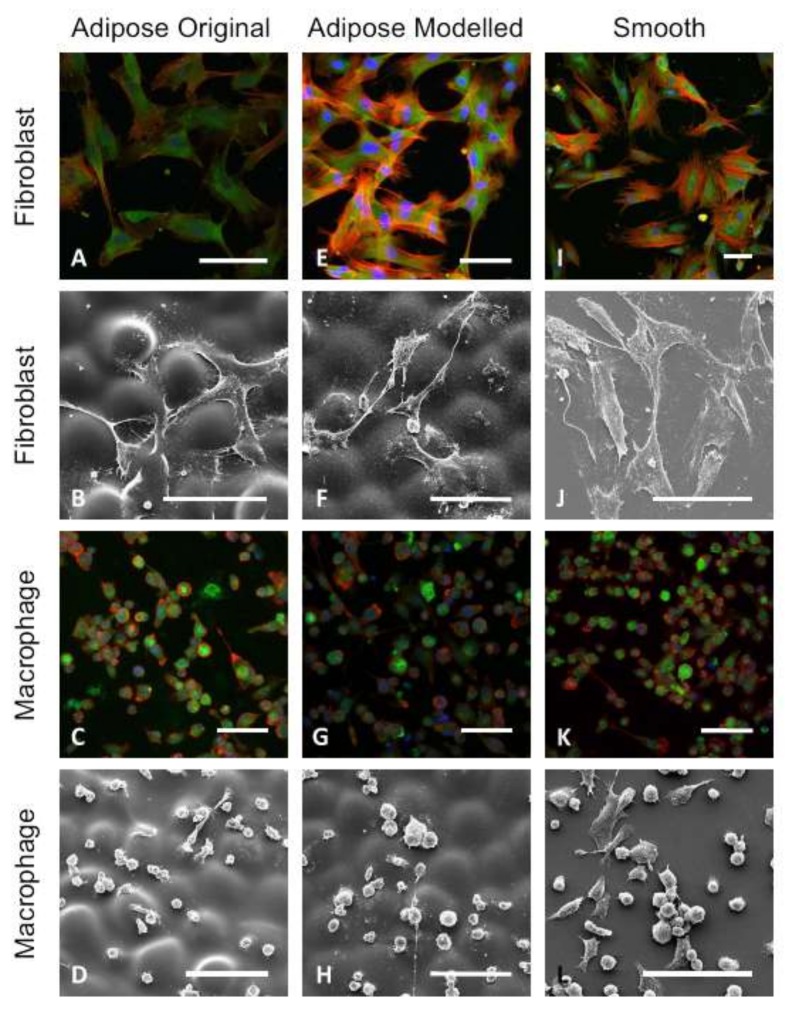
Immunocytochemistry and SEM images (scale bars = 100 µm). Fibroblasts and macrophages were grown in culture on (**A**–**D**) the original adipose; (**E**–**H**) modeled adipose; and (**I**–**L**) smooth control surfaces; (**A**,**E**,**I**) Few differences were observed in focal contacts and cells were well spread and had a classical spindle shape; (**B**) Fibroblasts on the original adipose surface aligned with valleys between the hemispherical shapes; (**F**) while cells on the modeled surface spread across the surface and their secondary texture masked the primary hemispherical nature; (**D**,**H**) Macrophages did not align with the underlying primary topography and adhered to the uppermost surfaces of the original and modeled adipose surfaces; (**D**,**H**,**L**) Macrophages cultured on the modeled adipose surfaces spread less than those on the original adipose and smooth surfaces [120].

Barr et al. [120] created biomimetic breast tissue-derived implant surfaces through three-dimensional grayscale photolithographic and oxygen plasma-etching techniques (Figure 9). Pro-inflammatory genes, such as those encoding *IL-β1*, *TNF-α*, and *IL-6*, were down-regulated and anti-inflammatory gene *IL-10* was up-regulated on the novel surfaces. Immunocytochemistry and SEM show the fibroblasts were well spread and spindle-shaped, and that macrophages had favorable responses to these novel surfaces (Figure 10). 

**Figure 11:**
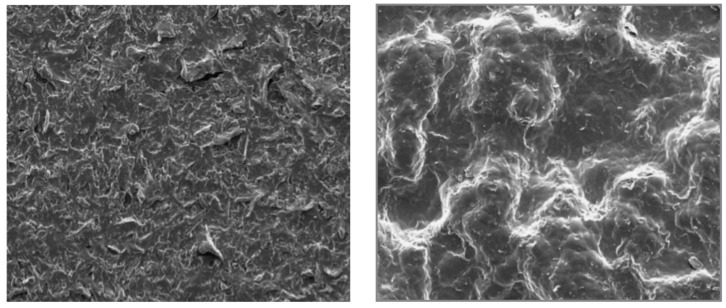
Nano- and microtextured surfaces visualized by SEM [121].

Sforza et al. [121] performed a preliminary 3-year evaluation of nano/microtextured silicone breast implants. In this study, the nano/microtextured breast implants were constructed with a uniform topography using three-dimensional imprinting of PDMS to create optimized biocompatible outer shells. Fabrication was particle-free and did not use extrusion of foreign material to create the surface geometry, thus enabling a uniform and controlled shell thickness (Figure 11) [121]. The physical characteristics of the nano/microtextured surfaces are presented in Table 1. The rate of complications, e.g., early seromas, infections, hematomas, wound dehiscence, ruptures, and implant malpositions, with nanotextured surface implants, was 0.36% (95% CI: 0.19 to 0.68%) compared to 1.06% (95% CI: 0.76 to 1.47%) with microtextured surface implants. However, the short-term nature of the study limits comparisons of capsular contracture formation and ALCL occurrence. Long-term studies are required.

## 4. Future Directions

Application of novel approaches from the fields of micro- and nanotechnology for the development of breast implants, such as micro- and nanotopography, could improve biointegration and enhance biocompatibility of implants. Surface modification of the nano/microtopographies by integrating reservoirs for controlled release of antimicrobial or anti-inflammatory agents into them could potentially reduce capsular contracture. Thus, the nanoscale architecture of the breast implants could be modified to alter the body’s immune response to them, minimize biofilm formation, and affect the subsequent degree of capsular contracture. This could lead to the development of permanently implantable materials with immunologically inactive nano-engineered surfaces. Furthermore, in the near future, nano/microelectromechanical devices and breast cancer cell-specific proteins integrated within newer implants could be used to detect cancer cells, cancer recurrence, and treat pathologic cells.

## 5. Conclusions

To date, a wide variety of studies has investigated the reduction of capsular contracture caused by silicone implants. Table 2 shows an overview of techniques for reducing implant-inducted contracture and associated foreign body responses. However, complications caused by silicone implants remain unresolved. Researchers are attempting to reduce these complications. Studies focusing on developing implant surfaces similar to human tissues are of particular importance. A physiological ECM-like surface reduces inflammatory foreign body reactions and modulates the immune response. The plastic and reconstructive surgery fields have significantly benefited from nano/microtechnology in cosmetic dermatological applications, wound healing, implant and prosthesis development, tissue engineering and regenerative medicine, and drug delivery materials. In particular, the development of materials has evolved due to nano/microtechnology that enables the analysis of material surface topography. Recently, a novel implant technology utilizing various nano-/micro-engineering techniques has emerged. Development of implants using these new technologies would allow increasingly natural interactions between the implants and surrounding tissues, which would reduce the peri-implant inflammatory response in the clinic and the induction of chronic inflammation in cells and tissues. Although no clinical long-term follow-up results on these implants have been reported, novel implant surfaces with improved interactions with surrounding tissues may reduce the risk of capsular contracture and should improve the results in anaplastic large cell lymphoma. In the field of plastic and reconstructive surgery, the nano/microtechnology that develops a more advanced biocompatible implant will continue to grow and expand, and continued research is needed on new biomaterials that mimic human tissues.

## Figures and Tables

**Table 1:** Physical characteristics of nano/microtextured surfaces.

Characteristics	Nanotextured	Microtextured
Consistent surface roughness	4000 nanometers on average (Ra)	17 ± 3 µm
Median profile height (µm)	13 ± 2	57 ± 15
Kurtosis	3.1 ± 0.4	2.6 ± 0.3
Skewness	0.4 ± 0.2	0.1 ± 0.2
Contact angle *	131° ± 4°	119° ± 3°
Contact points per cm^2^	49,000	1800–2200

* Contact angle reveals how the topography increases hydrophobicity compared to a smooth PDMS (surface contact angle of less than 110° ± 4°) [121].

**Table 2:** Overview of techniques for reducing implant-induced contracture and associated foreign body responses.

Type of Method	Subtype	References
Systemic drugs	Antibiotics	[34,35]
	Leukotriene antagonists	[36,37,38,39,40,41,42,43,44]
	Angiotensin-converting enzyme inhibitors	[45]
	Anti-fibrotics	[46,50]
	Colchicine	[51]
	Vitamin E	[54,58]
	Synthetic tryptophan metabolite	[59]
Topical application	Anti-adhesion agents	[77,78]
	Antibiotics	[66,67]
	Leukotriene antagonists	[78,79]
	Steroids	[81,82,83]
	Povidone-iodine	[64,65]
	5-Fluorouracil	[72]
	Type A Botulinum toxin	[68,69]
	Hyaluronidase	[70,71]
	Mitomycin C	[73]
	Collagenase	[76]
	Synthetic tryptophan metabolite	[80]
	Halofuginone	[84]
	Colchicine	[89]
	Vitamin E	[90]
	Croton oil	[90]
	Nicotine	[91]
	Medical chitosan	[92]
Materials	Combined with autologous tissue	[93,94,95,96,97]
	Combined with acellular dermal matrix	[98,100,101,102]
Surface types	Smooth and textured surfaces	[3,23,103,104,105,106,107,108,110,111]
	Nano-micro modifications	[112,113,114,115,116,117,118,119,120,121]

## Abbreviations

ALCL	Anaplastic large cell lymphoma
IL-17	Interleukin-17
IL-6	Interleukin-6
IL-8	Interleukin-8
TGF-β1	Transforming growth factor-β1
IFN-γ	Interferon-gamma
LTAs	Leukotriene antagonists
Cys-LT1	Cysteinyl leukotrienes receptors type 1
PFD	Pirfenidone
ADM	Acellular dermal matrix
ECM	Extracellular matrix
FCs	Human fibroblasts
ECs	Human endothelial cells
SMCs	Human smooth muscle cells
PDMS	Polydimethylsiloxane
SEM	Scanning electron microscopy
ADM PDMS F	Acellular dermal matrix polydimethylsiloxane fabricated surface
ADM PDMS C	Acellular dermal matrix polydimethylsiloxane cast surface
BDFs	Breast-derived fibroblasts
TNF-α	Tumor necrosis factor-alpha
